# COVID-19 related stress during and one year after the first wave of the pandemic outbreak in China: The role of social support and perceptions of the pandemic

**DOI:** 10.3389/fpsyt.2022.1009810

**Published:** 2022-12-01

**Authors:** Jingchu Hu, Jiayu Liu, Yiting Huang, Zhiying Zheng, Dongliang Yang, Yunfei Zhou, Jianhong Wang

**Affiliations:** ^1^Department of Anxiety Disorders, Shenzhen Mental Health Center, Shenzhen Kangning Hospital, Shenzhen, China; ^2^Shenzhen Mental Health Center, Shenzhen Kangning Hospital, Shenzhen, China; ^3^Cangzhou Medical College, Cangzhou, China

**Keywords:** COVID-19, stress, social support, perception, China

## Abstract

**Introduction:**

COVID-19 related stress might vary with the pandemic changes, as well as other associated factors. This study aimed to compare the stress level during the first wave of the pandemic outbreak and 1 year later in China, and to explore the differential roles of social support and perceptions of this disease in affecting pandemic-related stress over time.

**Methods:**

COVID-19 related stress, social support, and perceptions of the pandemic (perceived threat, perceived protection, and perceived controllability) were measured using the Impact of Event Scale-Revised for COVID-19, the Multidimensional Scale of Perceived Social Support, and the Self-Compiled Scale of COVID-19 Related Perception, respectively. Using an online survey, two independent samples were collected during the first wave of the COVID-19 outbreak (Time 1: March 2020, *N* = 430) and 1 year later (Time 2: April 2021, *N* = 512).

**Results:**

Levels of COVID-19 related stress and social support were lower at Time 2. Furthermore, at both Time 1 and Time 2, more social support was associated with less stress. Perceived protection and controllability of COVID-19 also mediated the relationship between social support and COVID-19 at both time points. However, the perceived threat of COVID-19 only served as a mediator at Time 1.

**Conclusion:**

These results indicate that Chinese people might experience lower COVID-19 related stress as the pandemic progresses. The perceived threat of COVID-19 played a more critical role in stress experienced at Time 1. These findings not only underscore the importance of social support under the context of Chinese society, but also have implications for developing specific interventions targeting different perceptions of COVID-19 to reduce pandemic-related stress during the different waves of this pandemic.

## Introduction

In late December 2019, China was the first country to identify the coronavirus disease (COVID-19) as the cause of a spreading pandemic. While COVID-19 has pervaded the narrative of 2020–2022, the virus is still novel and highly transmissible. This disaster has an inevitably long-term and negative impact on the mental health of the general public in China (1–3).

Previous literature suggests stress response is one of the most common mental health outcomes of pandemics (e.g., severe acute respiratory syndrome [SARS] and Ebola) (4–6). At the beginning of the COVID-19 outbreak, a cross-sectional study in China indicated that approximately one-quarter of the sample experienced acute stress reactions (7). Other studies have reported that COVID-19 causes stress responses (e.g., COVID-19 related intrusive thoughts) and affects people’s mental health and lifestyle habits (8–12). Although some studies have investigated the factors influencing COVID-19 related stress, such as coping strategies and chronic diseases (13, 14), only a few have compared levels of COVID-19 related stress across different time points. In addition, it remains unclear which factors and dynamics are associated with the stress responses induced by COVID-19.

Several studies have reported a negative relationship between social support and stress responses as an important factor that can buffer the latter (15, 16). However, little is known about the processes that underlie the links between social support and stress. Joseph et al. (17) proposed a model suggesting that social support relieves stress reactions by influencing people’s perceptions and interpretations of traumatic stressors. Recent studies have further indicated that perceptions of traumatic stressors have affected mental health during the COVID-19 outbreak (8, 18–20). For example, the perceived risk of COVID-19 is positively correlated with preventive health behaviors (21) and stress responses (22). Nevertheless, few researchers investigate the roles of COVID-19 related perceptions in the relationship between social support and pandemic-related stress. This may be attributed to the lack of corresponding measurements on the different COVID-19 perceptions (e.g., perceived threat and perceived controllability). Therefore, novel measures need to be developed to better understand the influence of COVID-19 perceptions herein. In addition, considering pandemic-induced lifestyle changes (e.g., the closure of gyms and universities), social support and COVID-19 perceptions might have differed during the different waves of the outbreak (11, 23). More research should be conducted to explore the relationships between perceptions of COVID-19, social support, and stress responses at different time points during the pandemic.

**FIGURE 1:**
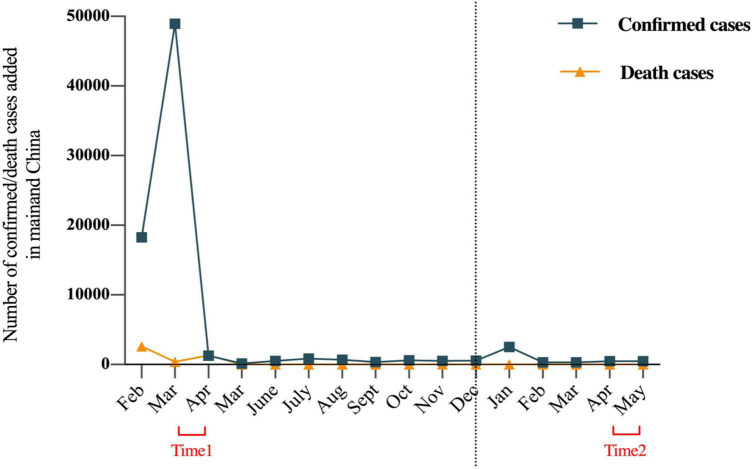
Trajectory of the COVID-19 in mainland China with the number of confirmed and deaths cases added each month from February 2020 to May 2021. The left side of the dotted line is 2020, and the right side is 2021.

This study aimed to compare levels of COVID-19 related stress in Chinese people during the first wave of the COVID-19 outbreak and 1 year later (Figure 1). In the current study, we tested three hypotheses: (1) compared to 1 year after the first wave of the COVID-19 outbreak, the level of COVID-19 related stress would be higher at Time 1, and social support would change between Times 1 and 2; (2) social support would negatively correlate with COVID-19 related stress in both periods; and (3) perceptions of COVID-19 mediate the association between social support and COVID-19 related stress in both periods.

## Materials and methods

### Study design

Data were collected using an anonymous cross-sectional online survey. Two time points were selected: March 2020 (Time 1: the first wave of the COVID-19 outbreak in China) and April 2021 (Time 2: 1 year later). A total of 942 participants were recruited (*N*_Time 1_ = 430, *N*_Time 2_ = 512). The study was approved by the institutional review board of Kangning Hospital. All participants provided informed consent prior to responding to the survey.

The survey was provided by the Chinese online platform www.wjx.cn and was anonymous to ensure data reliability and confidentiality. We also set up trap questions in the questionnaire to ensure answer quality. Participants included in the data analysis met the following criteria: (1) all questions were answered thoughtfully and (2) the trap question was answered correctly (e.g., What is the capital city of China?). Responses from participants who failed the trap question and who chose the same answers across the entire scale were deleted. Qualifying participants were all offered the same compensation.

### Instruments

#### The Impact of Event Scale-Revised Version

The Chinese version of the IES-R is a 22-item measure of stress reactions related to the COVID-19 pandemic (24). Each item describes the difficulty individuals sometimes have after experiencing a stressful COVID-19 event. Responses were rasted on a five-point Likert scale ranging from 0 (“not at all”) to 4 (“extremely”), which indicate the level of distress caused by COVID-19 during the past 7 days (the total scores range from 0 to 88, with higher scores indicating greater distress). Example items included, *“I tried not to think about COVID-19,” “I tried to remove COVID-19 from my memory,”* and *“I had dreams about COVID-19”* (for details, see Supplementary Table 1). The three dimensions of the scale were: (1) COVID-19-related intrusion, (2) avoidance, and (3) hyperarousal. This study focused on the total score, which ranged from 0 to 88. Considering that: (1) the survey was based on the past 7 days, which did not match the DSM-5 diagnosis for posttraumatic stress disorder (PTSD), and (2) though the data collection included two time points, the pandemic is still ongoing and, therefore, not a post-traumatic event. Consequently, the IES-R was conceptualized as a measure to assess COVID-19 related stress rather than PTSD symptoms in the current study (25). For the first phase of testing, during the outbreak, the Cronbach’s α coefficient for this scale was 0.85. After 1 year, at the second testing, the Cronbach’s α was again 0.85. Both indicate adequate reliability.

#### Multidimensional Scale of Perceived Social Support

The Chinese version of the MSPSS is a 12-item self-reported measure used to assess levels of social support from three sources: family, friends, and significant others (26). Participants rated their agreement on a seven-point Likert scale from 1 (“very strongly disagree”) to 7 (“very strongly agree”), with higher scores indicating higher perceived social support (total scores ranging from 12 to 84). Example items included, *“My family really tries to help me,” “I have a special person who is a real source of comfort to me,”* and *“I can count on my friends when things go wrong.”* The three dimensions in this scale were: family support, friend support, and other support. The Cronbach’s α coefficient was 0.89 for Time 1 and 0.90 for Time 2, indicating adequate reliability.

#### Self-Compiled Scale of COVID-19 Related Perception

The SSCP is a self-compiled and self-reported questionnaire containing ten items that is mainly used to assess individuals’ COVID-19 perceptions. All items were rated on a 7-point Likert scale, ranging from 1 (“strongly disagree”) to 7 (“strongly agree”). Exploratory and confirmatory factor analyses were used to determine the internal structure of the scale (total scores ranged from 10 to 70). Example items included, *“I think my life and health were threatened by COVID-19,” “I think wearing protective equipment (e.g., masks) can protect me from COVID-19,”* and *“I think the treatment for the virus is effective”* (for details, see Supplementary Table 2). The scale has three sub-dimensions: perceived threat, perceived protection, and perceived controllability. Cronbach’s alpha for this scale was 0.60 for both Time 1 and Time 2, indicating adequate reliability. The scale validity is further described in the Results section.

### Statistical analyses

Data analysis was performed using IBM SPSS statistical software (version 23.0; IBM Corp.) and Mplus 8.3. Statistical significance was set at *p* < 0.05 and all tests were 2-tailed. Only completed surveys were analyzed. To examine the reliability and validity of the SSCP, item analysis, exploratory factor analysis (EFA), and confirmatory factor analysis (CFA) were conducted using the sample from Time 1. Then, the demographic characteristics (e.g., age, gender, and income) were compared between the two samples from the two time points using the Pearson χ^2^ test. The scores for COVID-19 perceptions, COVID-19-related stress, and perceived social support at Times 1 and 2 were not normally distributed, nor were the distributions similar. The non-parametric Mann–Whitney *U* test was applied, with the mean rank presented. Finally, an analysis was performed to identify correlations between the psychological factors. The mediation analysis was conducted using the PROCESS 3.0 procedure with SPSS to examine the associations and mechanisms, with all the covariates being controlled.

## Results

### The Self-Compiled Scale of COVID-19 Related Perception’s internal structure and dimensionality

Item analysis was conducted with the participants from Time 1. The critical ratio method was used, with all participants being ranked according to their total scores from high to low. The independent-sample *t*-test results indicate that all items could be significantly discriminated and had good psychometric properties (*p* < 0.001).

Next, all items were used to conduct an EFA with participants from Time 1. Bartlett’s test of sphericity (χ^2^ = 777.26, *df* = 45, *p* < 0.001) and the KMO index = 0.718 indicate that the correlation matrix was suitable for factor analysis. The EFA of the scale produced three significant factors (Supplementary Table 3) with eigenvalues > 1 that explained 56.92% of the variance. The first and second factors contained three items each, whereas the third factor contained four items (the explained variances were 25.66, 20.09, and 11.18%, respectively). The three factors were labeled perceived threat perceived protection, and perceived controllability.

To substantiate the factor structure identified through the EFA, a CFA was conducted using Time 1 participants. The results indicate the structure of the SSCP with three factors, and 10 items had adequate good fit (χ^2^ = 46.67, *df* = 32, RMSEA = 0.03, CFI = 0.98, TLI = 0.97, SRMR = 0.03). Therefore, the SPSRC scale had good validity and was used in the subsequent analyses.

### Demographic characteristics

A total of 942 eligible participants from the two time points were included in the final analysis. Pearson’s χ^2^ test showed that the two participant groups differed significantly in age (*p* < 0.05), but not in gender, only-child family status, education level, income, or occupation (Table 1) (*ps* > 0.05).

**TABLE 1:** Demographic characteristics of participants of two periods.

Variables	Time 1 (*N* = 430)	Time 2 (*N* = 512)	Time 1 vs. Time 2
		
	No. (%)		*P*-value^a^
Sex			
Male	215 (50)	228 (44.5)	0.094
Female	215 (50)	284 (55.5)	
Whether in only-child family			
Yes	162 (37.7)	168 (32.8)	0.119
No	268 (62.3)	344 (67.2)	
Age, years			
<20	44 (10.2)	26 (5.1)	0.031
20–29	216 (50.2)	277 (54.1)	
30–39	130 (30.2)	163 (31.8)	
40–49	32 (7.4)	41(8.0)	
50–59	8 (1.9)	5 (1.0)	
Education			
≤Junior high school	7 (1.6)	4 (0.8)	0.733
Senior high school	36 (8.4)	40 (7.8)	
College	79 (18.4)	101 (19.7)	
Undergraduate	290 (67.4)	342 (66.8)	
≥Postgraduate	18 (4.2)	25 (4.9)	
Household income, yuan			
<50,000	51 (11.9)	42 (8.2)	0.130
50,000–100,000	133 (30.9)	137 (26.8)	
100,000–200,000	150 (34.9)	193 (37.7)	
200,000–500,000	85 (19.8)	123 (24.0)	
500,000–1,000,000	8 (1.9)	15 (2.9)	
>1,000,000	3 (0.7)	2 (0.4)	
Career			
Worker	25 (5.8)	49 (9.6)	0.122
Farmer	8 (1.9)	2 (0.4)	
Student	99 (23)	105 (20.5)	
Medical staff	10 (2.3)	13 (2.5)	
Educational, scientific and cultural personnel	25 (5.8)	26 (5.1)	
Enterprise manager	171 (39.8)	193 (37.7)	
Government institution personnel	36 (8.4)	54 (10.5)	
Retiree	1 (0.2)	0 (0)	
Migrant worker	22 (5.1)	21 (4.1)	
Other	33 (7.7)	49 (9.6)	

COVID-19, coronavirus disease 2019. ^a^Two-tailed χ^2^ analysis conducted for significance testing.

### COVID-19 related stress, social support, and perceptions of the COVID-19

**FIGURE 2:**
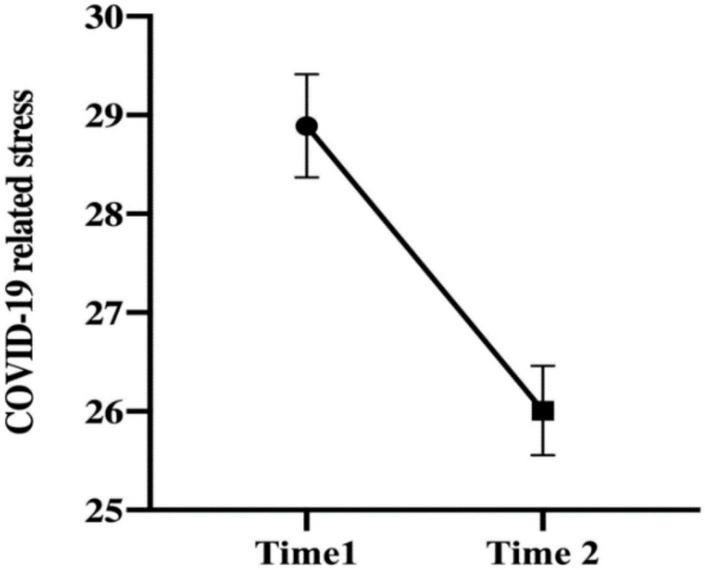
Mean score of the COVID-19 related stress (IES-R) at two time points. Time 1, first wave of the outbreak; Time 2, 1 year after the first wave. Error bars indicate SEs.

Mann–Whitney *U* test results indicate a significant difference in stress at Times 1 and 2 (*p* < 0.001). Considering mean rank cannot be visually compared in the figure, both the mean rank (Table 2) and means suggested that stress levels were lower after 1 year. Figure 2 depicts this decreasing trend after 1 year with means and standard errors. The levels of intrusion (*p* < 0.001) and avoidance (*p* = 0.001) were significantly lower at Time 2 (*p* < 0.05). Although there was no significant change in perceived social support, participants reported a slightly higher level of perceived support during the pandemic period (*p* = 0.156). SSCP analysis indicated that perceived threat (*p* < 0.001) and perceived protection (*p* < 0.001) were lower after 1 year, whereas the sense of controllability (*p* = 0.02) was higher (Table 2).

**TABLE 2:** Self-reported scores during the first wave of COVID-19 and 1 year later.

	Time 1	Time 2	Time 1 vs. Time 2
		
	Mean rank		*P*-value
Perceived threat of COVID-19	512.90	436.73	< 0.001
Perceived protection of COVID-19	515.26	434.75	< 0.001
Perceived controllability of COVID-19	449.18	490.25	0.02
COVID-19 related stress (IES-R)	514.38	435.48	< 0.001
Intrusion	514.87	435.08	< 0.001
Avoidance	504.07	444.15	0.001
Hyperarousal	490.14	455.85	0.053
MSPSS	485.22	459.98	0.156

COVID-19, coronavirus disease 2019; IES-R, 22-item Impact of Event Scale-Revised; MSPSS, Multidimensional Scale of Perceived Social Support.

### Correlation and mediation analysis

Bivariate correlation analysis results indicated that social support was negatively correlated with COVID-19 related stress at Times 1 and 2 (Supplementary Table 4). Further, the three dimensions of COVID-19 related perceptions were also significantly correlated with social support and stress at Time 1 (Supplementary Table 4). One year later, perceived protection and perceived controllability remained significantly correlated with social support and COVID-19 related stress (Supplementary Table 4). However, perceived threat was not significantly correlated with social support (Supplementary Table 4). As a result, perceived threat was not analyzed for Time 2.

**FIGURE 3:**
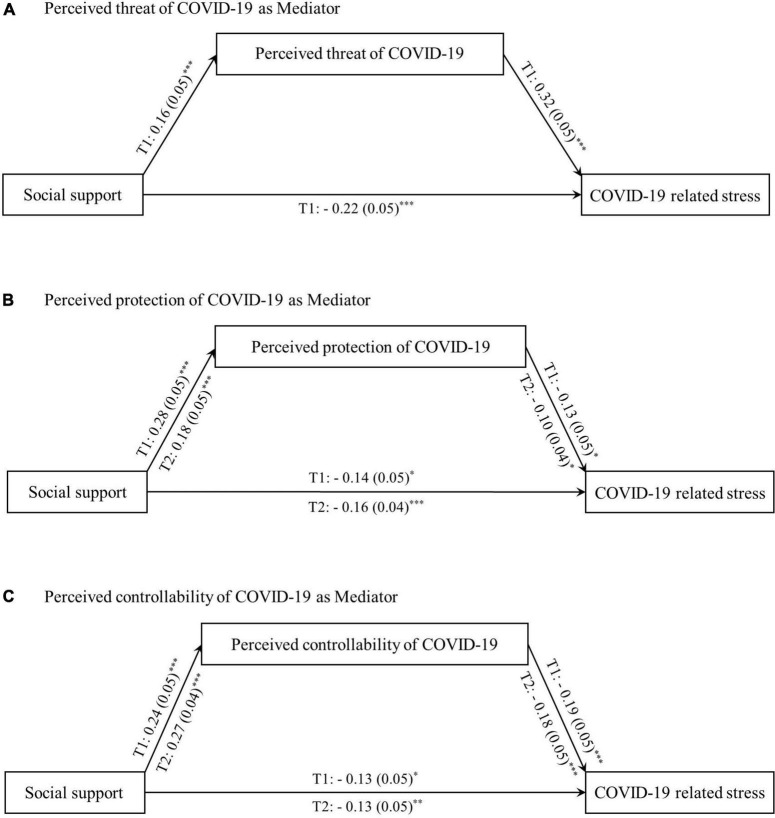
The mediation of perceived threat, perceived defense, and perceived controllability during the first wave of the COVID-19 outbreak and one year later. The link between social support and COVID-19 related stress is mediated. Path values are the path coefficients (standard errors). All covariates (whether in one-child family, education, age, gender, career, and income) were controlled in the analysis. T1 is the first wave of the outbreak; T2 is the second wave of 1 year after the first wave. **(A)** Shows that the perceived threat of COVID-19 mediates the relationship between social support and COVID-19 related stress in T1, but not in T2. **(B)** Shows that the perceived protection of COVID-19 mediates the relationship between social support and COVID-19 related stress both in T1 and T2. **(C)** Shows that the perceived controllability of COVID-19 mediates the relationship between social support and COVID-19 related stress both in T1 and T2. *p < 0.05, **p < 0.01, and ***p < 0.001.

Mediation analyses were performed individually, with participants from Times 1 and 2. All continuous variables were standardized to a mean of 0 and a standard deviation of 1 before the analyses to facilitate interpretation of the main and mediation effects. In the analysis of data from Time 1, after controlling for demographic variables, three dimensions of perceptions of COVID-19 significantly mediated the association between social support and stress. First, perceived threat significantly mediated this association (95% CI, 0.01–0.10) (Figure 3A). Nevertheless, this indirect mediation effect was inconsistent with the direct effect, with perceived threat working as a suppressed mediator (11). Moreover, social support was negatively associated with COVID-19 related stress (β = −0.22; *p* < 0.001). However, perceived threat was positively associated with social support (β = 0.16; *p* < 0.001) and stress (β = 0.32; *p* < 0.001). Consequently, perceived threat partially explained the relationship between social support and COVID-19 related stress.

Next, perceived protection partially mediated the association between social support and COVID-19 related stress (95% CI, −0.07 to −0.003), with an estimated 21.05% (Figure 3B). Specifically, social support was positively associated with perceived protection (β = 0.28; *p* < 0.001). However, it was negatively associated with stress (β = −0.13; *p* < 0.05). Similarly, perceived protection was negatively associated with COVID-19 related stress (β = −0.14; *p* < 0.05). Moreover, perceived controllability partially mediated this association (95% CI, −0.08 to −0.02), with an estimated 27.18% (Figure 3C). Social support was positively associated with perceived controllability (β = 0.24; *p* < 0.001) but negatively associated with stress (β = −0.13; *p* < 0.05). Similarly, perceived controllability was negatively associated with COVID-19 related stress (β = −0.19; *p* < 0.001).

One year after the first wave of the pandemic (Time 2), only perceived protection and controllability had a mediating effect on the association between social support and COVID-19 related stress. Perceived threat did not correlate with social support and did not act as a mediator. After controlling for demographic variables, an estimated 10% of the association was mediated through perceived protection (95% CI, −0.04 to −0.001) (Figure 3B). Social support was positively associated with perceived protection (β = 0.18; *P* < 0.001) but negatively associated with stress (β = −0.16; *p* < 0.001). Perceived protection was also negatively associated with COVID-19 related stress (β = −0.10; *p* < 0.05). Similarly, perceived controllability partially mediated this association (95% CI, −0.08 to −0.02), with an estimated 26.82% (Figure 3C). Social support was found to be positively associated with perceived controllability (β = 0.27; *p* < 0.001) but negatively associated with stress (β = −0.13; *p* < 0.01). In addition, perceived controllability was negatively associated with COVID-19 related stress (β = −0.18; *p* < 0.001).

## Discussion

This study investigated the relationships between COVID-19 related stress, social support, and perceptions of COVID-19 during different waves of the pandemic in China. The results found support for our hypotheses. Stress levels were lower 1 year after the first wave of the pandemic outbreak. Moreover, the relationship between social support and stress was mediated by perceived protection and perceived controllability in both Times 1 and 2. However, perceived threat was a mediator only at Time 1. These findings provide new evidence of the pandemic’s temporal changes in China and improves current understandings of the psychological mechanisms underlying these trends.

Our results revealed a similar decrease in COVID-19 related stress to that of a United States longitudinal study, which supports our first hypothesis (27). These findings might be due to the age range (about 90% under 40 years old) and jobs (about 50% are enterprise managers or students) of our sample. Most of our participants were young and healthy. They usually received more social support and better adapted to stress (12–14). However, some researchers have found a contrary tendency (28, 29). One possibility for this is the relatively low number of positive COVID-19 cases at Time 2 in China, which may explain why people experienced fewer stress reactions. An alternative explanation is that quite a few Chinese cities were in lockdown during Time 1. This sudden lifestyle change could have increased stress levels (30), which would have decreased after the cities reopened (31).

In contrast to recent findings showing higher social support as the lockdown was lifted (32), we found that people reported lower social support 1 year after the first wave of the pandemic. This deviation from expectation may be partly due to post-pandemic changes in people’s lifestyles and jobs (e.g., more people preferring to work from home or losing their jobs) (33). It is also worth noting that our results suggest that social support as a protective factor is significantly and negatively correlated with COVID-19 related stress across different time points. This is in line with our second hypothesis and previous studies (2, 34).

Further, as with our third hypothesis, perceived protection and perceived controllability mediated the association between social support and stress at both Times 1 and 2. Previous research has suggested that support from the government, family, and friends influences people’s perceived risk and health-seeking behaviors (35). Concurrently, a higher level of perceived safety and sense of control consequently alleviates stress responses (36, 37). Equally important, social support indirectly influenced COVID-19 related stress through the perceived threat of COVID-19 during the outbreak. However, this meditation effect was not observed after 1 year, indicating that the perceived threat only had a conditional impact on the association under the special pandemic circumstances. Noticeably, perceived threat acted as a suppressor of the mediating effect of social support on stress. In line with these results, social support may not always be coping mechanism for distress (37). In a distressing environment, people may not want to be exposed to greater concerns or unwanted information from social contacts, which can lead to uncertainty and anxiety (38). Therefore, at Time 1, when most people faced numerous struggles (e.g., in finance, work, and mental health), social support may have reinforced their negative feelings. These findings provide new evidence for the influence of social support on COVID-19 related stress and insights into the importance of social support on mental health during the pandemic in the current society of China.

### Strengths and limitations

The current study extended our previous work (39) by examining the perceptions of COVID-19 as mediators in the mechanism of social support influencing COVID-19 related stress. Adequate social support provides individuals with more information on COVID-19, thus reducing COVID-19 related stress and promoting mental well-being. In addition, these findings provide insights into interventional strategies for mental well-being. Interestingly, perceived threat had a suppressive mediation effect, which might mean that under special circumstances (i.e., highly contagious infectious situations), social contact may increase perceived threat, thus affecting well-being and health. This study does have several limitations. First, owing to the cross-sectional nature of the design, causal inferences could not be made. Further experimental research is required to confirm these relationships. Second, the online data collection method used may have affected the survey reliability. Future studies should also consider using other measurements. Third, most of the participants in this study were managers and students; therefore, caution should be exercised in generalizing the present results to people with other jobs.

## Conclusion

In summary, at Time 2, Chinese people reported less COVID-19-related stress and social support. Furthermore, perceived protection and controllability of COVID-19 mediated the relationship between social support and stress at Times 1 and 2. The perceived threat of COVID-19 only functioned as a mediator during the first wave. These results indicate that the stress response may fluctuate over time. The perceived threat of COVID-19 seemed to play a more important role between social support and stress at the beginning of the outbreak. Future research is needed to examine and address potential disparities in COVID-19 related stress and social support over time. Public health interventions should emphasize the importance of modulating perceptions of COVID-19 over the pandemic course. In addition, the use of technology in facilitating social support during the pandemic should be explored.

## Data availability statement

The datasets presented in this study can be found in the Supplementary material and online repositories: https://osf.io/g2eup/.

## Ethics statement

The studies involving human participants were reviewed and approved by Institutional Review Board of Kangning Hospital. The patients/participants provided their written informed consent to participate in this study.

## Author contributions

JH and JL: designed the study. YH, ZZ, and YZ: participated in the data collection. YH and JL: analyzed the data. JH and DY: advised on methodology. JH, JL, and YH: drafted the manuscript. JH, YZ, and JW: edited the manuscript and supervised the data collection. All authors contributed to the article and approved the final manuscript.
