# Supplementary material for: COVID-19 related stress during and one year after the first wave of the pandemic outbreak in China: The role of social support and perceptions of the pandemic
Source: Front Psychiatry. 2022 Dec 1;13:1009810. doi: 10.3389/fpsyt.2022.1009810 (PMC9751691; doi:10.3389/fpsyt.2022.1009810)
Supplement: Supplementary file 1 [file Data_Sheet_1.docx]

TABLE S1.

*Impact of Events Scale-Revised (IES-R) for COVID-19.*

| Instructions:  Below is a list of items that describe. For each item, please rate how distressing each difficulty has been for you DURING THE PAST SEVEN DATS with respect to COVID-19. | 0 | 1 | 2 | 3 | 4 |
| --- | --- | --- | --- | --- | --- |
| 1. Any reminder brought back feelings about COVID-19. |  |  |  |  |  |
| 1. I had trouble staying asleep. |  |  |  |  |  |
| 1. Other things kept making me think about COVID-19. |  |  |  |  |  |
| 1. I felt irritable and angry. |  |  |  |  |  |
| 1. I avoided letting myself get upset when I thought about it or was reminded of COVID-19. |  |  |  |  |  |
| 1. I thought about COVID-19 when I didn't mean to. |  |  |  |  |  |
| 1. I felt as if COVID-19 hadn't happened or wasn't real. |  |  |  |  |  |
| 1. I stayed away from reminders of COVID-19. |  |  |  |  |  |
| 1. Pictures about COVID-19 popped into my mind. |  |  |  |  |  |
| 1. I was jumpy and easily startled. |  |  |  |  |  |
| 1. I tried not to think about COVID-19. |  |  |  |  |  |
| 1. I was aware that I still had a lot of feelings about COVID-19, but I didn't deal with them. |  |  |  |  |  |
| 1. My feelings about COVID-19 were kind of numb. |  |  |  |  |  |
| 1. I found myself acting or feeling like I was back at that time. |  |  |  |  |  |
| 1. I had trouble falling asleep. |  |  |  |  |  |
| 1. I had waves of strong feelings about COVID-19. |  |  |  |  |  |
| 1. I tried to remove COVID-19 from my memory. |  |  |  |  |  |
| 1. I had trouble concentrating. |  |  |  |  |  |
| 1. Reminders of COVID-19 caused me to have physical reactions, such as sweating, trouble breathing, nausea, or a pounding heart. |  |  |  |  |  |
| 1. I had dreams about COVID-19. |  |  |  |  |  |
| 1. I felt watchful and on-guard. |  |  |  |  |  |
| 1. I tried not to talk about COVID-19. |  |  |  |  |  |

*Note.* This scale offers 5 different answer options which include options: Not at all, A little bit, Moderately, Quite a bit, Extremely.

TABLE S2.

*Self-compiled Scale of COVID-19 related Perception (SSCP).*

| Instructions:  Below is a list of items that describe. For each item, please rate how true you think it is for you. | 1 | 2 | 3 | 4 | 5 | 6 | 7 |
| --- | --- | --- | --- | --- | --- | --- | --- |
| 1. I think it is possible that I will recover after being infected with the virus. |  |  |  |  |  |  |  |
| 1. I think that my life and health was threatened by COVID-19. |  |  |  |  |  |  |  |
| 1. I think that my work/school was negatively affected by COVID-19. |  |  |  |  |  |  |  |
| 1. I think that the population in my area was threatened by COVID-19. |  |  |  |  |  |  |  |
| 1. I think that wearing protective equipment (e.g., masks) is protective for COVID-19. |  |  |  |  |  |  |  |
| 1. I think that strengthening personal hygiene (e.g., hand washing) is protective for COVID-19. |  |  |  |  |  |  |  |
| 1. I think that reducing contact with others (e.g., public transportation) is protective for COVID-19. |  |  |  |  |  |  |  |
| 1. I think that the transmissive path of the virus is well controlled. |  |  |  |  |  |  |  |
| 1. I think that the treatment for the virus is effective. |  |  |  |  |  |  |  |
| 1. I think that the local epidemic can be effectively controlled. |  |  |  |  |  |  |  |

*Note.* This scale offers 7 different answer options related to an agreement which include options: strongly disagree, disagree, somewhat disagree, either agree or disagree, somewhat agree, agree and strongly agree.

TABLE S3.

*Pattern matrix of the PCA for the self-compiled scale of COVID-19 related perception (SSCP).*

| Item | | Factor loading | | |
| --- | --- | --- | --- | --- |
|  |  | A | B | C |
| 1. Perceived threat of COVID-19 | 2. I think that my life and health  was threatened by COVID-19. | 0.747 |  |  |
|  | 3. I think that my work/school was  negatively affected by COVID-19. | 0.784 |  |  |
|  | 4. I think that the population in my area  was threatened by COVID-19. | 0.823 |  |  |
| 1. Perceived protection of COVID-19 | 5. I think that wearing protective  equipment (e.g., masks) is protective  for COVID-19. |  | 0.795 |  |
|  | 6. I think that strengthening personal  hygiene (e.g., hand washing) is  protective for COVID-19. |  | 0.790 |  |
|  | 7. I think that reducing contact with  others (e.g., public transportation) is  protective for COVID-19. |  | 0.700 |  |
| 1. Perceived controllability of COVID-19 | 1. I think it is possible that I will  recover after being infected with  the virus. |  |  | 0.719 |
|  | 8. I think that the transmissive path of  the virus is controlled. |  |  | 0.678 |
|  | 9. I think that the treatment for the virus  is effective. |  |  | 0.674 |
|  | 10. I think that the local epidemic can  be effectively controlled. |  |  | 0.624 |

TABLE S4.

*Correlations of all variables at Time 1 and Time 2.*

| Variables | Correlations | | | | |
| --- | --- | --- | --- | --- | --- |
|  | 1 | 2 | 3 | 4 | 5 |
| Time 1 | | | | | |
| 1.T1 Perceived Threat | - |  |  |  |  |
| 2.T1 Perceived Protection | 0.10^*^ | - |  |  |  |
| 3.T1 Perceived Controllability | -0.08 | 0.31^**^ | - |  |  |
| 4.T1 Social Support | 0.14^**^ | 0.31^**^ | 0.28^**^ | - |  |
| 5.T1 COVID-19 Related Stress | 0.22^**^ | -0.13^**^ | -0.18^**^ | -0.20^**^ | - |
| Time 2 | | | | | |
| 1.T2 Perceived Threat | - |  |  |  |  |
| 2.T2 Perceived Protection | 0.12^**^ | - |  |  |  |
| 3.T2 Perceived Controllability | -0.13^**^ | 0.29^**^ | - |  |  |
| 4.T2 Social Support | 0.02 | 0.21^**^ | 0.28^**^ | - |  |
| 5.T2 COVID-19 Related Stress | 0.22^**^ | -0.12^**^ | -0.23^**^ | -0.18^**^ | - |

*Note*. N_Time1_ = 430; N_Time2_ = 512.

^*^*p*< 0.05.^**^*p* <0.01.^***^*p* <0.001.
